# Anthelmintics efficacy against intestinal strongyles in horses of Sardinia, Italy

**DOI:** 10.1016/j.parepi.2016.01.001

**Published:** 2016-03-08

**Authors:** G. Sanna, A.P. Pipia, C. Tamponi, R. Manca, A. Varcasia, D. Traversa, A. Scala

**Affiliations:** a Dipartimento di Medicina Veterinaria, Settore di Parassitologia e Malattie Parassitarie, Università degli Studi di Sassari, Via Vienna 2, 07100 Sassari, Italy; b Veterinary practitioner, Sassari, Italy; c Facoltà di Medicina Veterinaria, Università degli Studi di Teramo, Piazza A. Moro 45, 64100 Teramo, Italy

**Keywords:** Anthelmintic treatment, Efficacy, Horses, Intestinal strongyles, Resistance

## Abstract

Intestinal strongyles (IS) are the most important parasites of equids, due to their high prevalence worldwide, pathogenicity and the spread of drug-resistant populations. Despite the large number of horses bred in Sardinia Island, Italy, no data are available on the efficacy of anthelmintic compounds in the control of horse strongylosis. Therefore the aim of the present study was to evaluate the efficacy of five commercial anthelmintic formulations containing fenbendazole (FBZ), pyrantel (PYR), moxidectin (MOX) and two ivermectin formulations (IVM1 and IVM2) against IS in Sardinia by performing a fecal egg count reduction test (FECRT) and investigating the egg reappearance period (ERP) after treatment. In total, 74 horses from 7 farms were examined. Coprocultures performed for individual fecal samples collected at the day of the treatment revealed that cyathostomins were the predominant parasitic species (98.6%). The FECR for all horses belonging to the treatment groups after two weeks was ≥ 95% with a 95% C.I. > 90%. The expected ERP did not decrease in any of the treatment group as FECR values < 90% were found at D60 for FBZ, at D90 for PYR and IVM1, at D150 for IVM2. All horses treated with MOX showed FECRT > 90% for the entire duration of the trial until D150. The results of the present survey indicate that drug-resistant cyathostomin populations are not present in the examined horse population, contrariwise to what observed in other Italian and European regions. The reasons and implications of these results are discussed.

## 1 Introduction

The most common and pathogenic parasitic nematodes of horses are members of the family Strongylidae, which includes large (Strongylinae Subfamily) and small (Cyathostominae Subfamily) strongyles. Intestinal strongyles (IS) of horses have a primary importance in equine medicine, for their worldwide distribution and impact on health and performance of infected animals (Heidi and Wade, 2009). In particular, small strongyles, also known as cyathostomins, are regarded as the most important helminth parasites of horses for their cosmopolitan diffusion and their pathogenic potential at both larval and adult stages (Peregrine et al., 2014). Currently, the control of IS infections is based on the use of systematic anthelmintic treatments and, when applied, on management measures, e.g. removal of feces from paddocks and grazing rotation (von Samson-Himmelstjerna, 2012, Nielsen et al., 2014, Papini et al., 2015). The abuse of anthelmintic for a long time in the so-called “blanket treatments” approach has caused a worldwide distribution of anthelmintic resistance (AR) in cyathostomin populations (Heidi and Wade, 2009, von Samson-Himmelstjerna, 2012, Nielsen et al., 2014). In particular, resistance to BZ such as fenbendazole (FBZ) is common and widespread, and increasingly reported for tetrahydropyrimidines, such as pyrantel salts (PYR). Macrocyclic lactones (MLs), i.e. ivermectin (IVM) and moxidectin (MOX), are still the most effective compounds against cyathostomins, despite reduced efficacy has been reported in both Europe and the Americas, especially for IVM (Traversa et al., 2009, Lyons et al., 2011, Molento et al., 2012, Traversa et al., 2012). In Italy, cyathostomins are widespread in horse populations, with prevalence up to 100% (Traldi et al., 1988, Piergili-Fioretti et al., 2005, Pilo et al., 2012); resistance to BZ, PYR and IVM has been reported in Italy (Traversa et al., 2009, Genchi et al., 1992, Traversa et al., 2007), with reduction of the Egg Reappearance Period (ERP) of IVM and MOX in few premises (Geurden et al., 2014). The evaluation of the ERP is crucial in understanding a possible onset of reduced efficacy in these parasiticides, especially for macrocyclic lactones (Sangster, 1999). It is worthy of note that the treatment efficacy in most studies is evaluated through a fecal egg count reduction test (FECRT) at up to 21 days after treatment. This approach might underestimate a potential reduction in anthelmintic efficacy of MLs, as reduced activity of IVM and MOX is initially apparent from a shortened ERP after treatment, and not necessarily from a reduced efficacy within few weeks after treatment (Lyons et al., 2011).

Despite the large number of horses bred in Sardinia island of Italy (17,066 heads, representing 4.3% of the national heritage 395,913), no reliable data are available on the efficacy of anthelmintics in this area (Montinaro et al., 2002). Therefore, the present study aimed at updating current knowledge on the efficacy and ERP of five commercial anthelmintic formulations commonly used for the control of IS in horses bred in Sardinia.

## 2 Materials and methods

A total of 74 horses, 40 females and 34 males ranging from 6 months to 6 years old from 7 premises in Sardinia were examined in 2010 for the presence of IS infection. All the tested animals were born and bred in Sardinia.

No anthelmintic treatments were performed in selected animals in the three months before the trial. In these farms breeders usually performed anthelmintic treatments once a year in the horses kept grazing and in paddock, while every 6 months in those animals living in boxes. All horses enrolled in the trial were kept under the same original farming conditions until the end of the study. Five different anthelmintics (oral paste/gel) licensed in Italy were evaluated; these were the most used in breedings in Sardinia: FBZ [Panacur® pasta, MSD], PYR [Strike®, os pasta Acme], two containing ivermectin (IVM1) [Eqvalan® os pasta Merial], (IVM2) [Eraquell® os pasta Virbac] and MOX [Equest® os gel Zoetis]. Animals were selected and randomly enrolled in one of the five treatment groups according to the number of horses present in each farm as shown in Table 1. Treatment was administered per os on Day (D) 0 after the estimation of the weight of each horse using a girth tape (Pavo and Virbac Horse Weight Tape®). All horses were observed for at least one hour after treatment to ensure complete swallowing of the drug and to record any side effects (e.g., sweating, mydriasis, colics). All the treatments were administered by the same person and laboratory personnel did not know to which group the sample belonged. The individual FECs of each horse was determined at day-7 prior to the FECRT, and then weekly in the first month after treatment (i.e., D7, D14, D21, D28), and thereafter, biweekly for the following 4 months (i.e., D45, D60, D75, D90, D105, D120, D135, D150) using McMaster slides according to Raynaud (Raynaud, 1970). The FECRT was carried out according to the recommendations of the World Association for the Advancement of Veterinary Parasitology (WAAVP) for detection of anthelmintic resistance in horses (Coles et al., 1992). Percentage reductions in the fecal egg count were determined for each treatment group with the following formula: 100 × (1 − post-treatment EPG arithmetic mean/pre-treatment EPG arithmetic mean).

**Table 1:** Anthelmintics evaluated for efficacy against horse strongyles in a population of horses from Sardinia Island, Italy.

Group	Horses enrolled	Commercial name	Company	Molecule	Administration	Dose
IVM1	15	Eqvalan®	Merial	Ivermectin	Oral	0.2 mg/kg BW
IVM2	17	Eraquell®	Virbac	Ivermectin	Oral	0.2 mg/kg BW
MOX	10	Equest®	Zoetis	Moxidectin	Oral	0.4 mg/kg BW
PYR	19	Strike®	Acme	Pyrantel pamoate	Oral	6.6 mg/kg BW
FBZ	13	Panacur®	MSD	Fenbendazole	Oral	7.5 mg/kg BW

The cut-off limits for establishing appropriate efficacy were arithmetic means FECR of 95% for MLs and > 90% for FBZ and PYR (Kaplan and Nielsen, 2010) and a 95% confidence interval (CI) of efficacy was calculated. The results of the FECRs were interpreted for all the compounds as follows: (1) resistance present if FECR < 90% for FBZ and PYR, ≥ 95% for MLs and the lower 95% confidence limit (LCL) < 90%, (2) resistance suspected if FECR ≥ 90% for FBZ and PYR, ≥ 95% for MLs and/or LCL < 90% and (3) no resistance if FECR ≥ 90% for FBZ and PYR, ≥ 95% for MLs and LCL > 90%.

The evaluation of the egg reappearance period (ERP) was also assessed, being defined as the time between treatment and the first breaching of a 90% efficacy threshold (Geurden et al., 2014) and based on the group arithmetic mean FEC. The standard minimum ERP used were 42 days for PYR, 42–56 days for FBZ, 56–70 days for IVM and > 91 days for MOX (Stratford et al., 2011).

Coprocultures were performed at D0 for each fecal sample and up to D150 in horses with EPG ≥ 150, in order to allow the in vitro growth of third stage strongyle larvae (L3) (Roberts and O'Sullivan, 1950), that were thereafter identified using the morphological keys by MAFF (MAFF (Ministry of Agriculture Fisheries and Food), 1986).

## 3 Results

No significant differences between treatment groups were found at D0 (P > 0.05), with mean EPG values ranging from 997.5 ± 493.92 to 1906 ± 1516.8 (Table 2). The FECR values at D14 in each treatment group constantly showed a ≥ 95% reduction with the 95% C.I. > 90%. In particular in IVM1 group the efficacy was 100% on D14 and until D75 in the MOX group (Table 2). FBZ group exceeded the ERP parameter at D60 (86% efficacy), the groups treated with PYR and IVM1 at D90 (88.7% and 87% respectively), group treated with IVM2 at D150 (87.7%) while group MOX for all the duration of the trial presented FECR efficacy > 90% (94% at D150) (Table 2). Larvae harvested at coprocultures carried out on samples collected at D0 were identified as Cyathostominae (96.4%), *Oesophagodontus* spp. (1.5%), *Strongylus vulgaris* (1.3%), *Gyalocephalus capitatus* (0.4%), *Trichostrongylus axei* (0.3%), *Strongylus equinus* (0.1%), *Triodontophorus* spp. (0.01%), and *Strongylus edentatus* (0.01%). All larvae recovered from coprocultures performed on post-treatment samples with values ≥ 150 EPG were identified as cyathostomins. No side effects were recorded throughout the study in the treated animals.

**Table 2:** Horse strongyles egg per gram (EPG) mean values, percentage of fecal egg count reduction (in brackets) and standard deviation (SD) in a population of horses from Sardinia Island, Italy investigated for the efficacy of five commercial formulations from Day 0 (D0) to Day 150 (D150).

Drug		D0	D7	D14	D21	D28	D45	D60	D75	D90	D105	D120	D135	D150
IVM 1	Mean EPG	1906	0 (100%)	0 (100%)	1 (99.9%)	5.36 (99.7%)	0 (100%)	16.2 (99.3%)	158.7 (92.8%)	295.9 (87%)	279.4(85.3%)	403.1(79.4%)	582.9 (75.8%)	1112.5 (57.3%)
	SD	1516.8	0	0	3.8	20.0	0	31.6	260.1	419.4	390.9	496.4	651.6	1246.7
IVM 2	Mean EPG	1504.4	6.2 (99.6%)	8.8 (99.4%)	6.2 (99.6%)	0 (100%)	0 (100%)	29.1 (98.1%)	157 (90.5%)	141.4 (91.1%)	64.5 (93.3%)	57.9 (91.3%)	38.6 (90.8%)	60 (87.7%)
	SD	1531.4	25.5	36.4	25.4	0	0	90.2	371.4	248.6	106.3	45.3	72.4	38.2
MOX	Mean EPG	997.5	0 (100%)	0 (100%)	0 (100%)	0 (100%)	0 (100%)	0 (100%)	0 (100%)	13.3 (98.8%)	42.9 (96.8%)	0 (97%)	18 (95.8%)	45 (94%)
	SD	493.9	0	0	0	0	0	0	0	40	106.9	0	40.2	90
PYR	Mean EPG	1405.3	15.8 (98.9%)	15.8 (98.9%)	30 (97.9%)	21.7 (96%)	46.8 (93.7%)	92.8 (93.7%)	135 (91.1%)	167.8 (88.7%)	236.8 (84.7%)	351.2 (78.1%)	242.1 (77.9%)	537.5 (70.5%)
	SD	1043.8	19.7	53.4	38.2	23.7	58.3	142.7	127.3	189.7	195.1	391.7	196	440.6
FBZ	Mean EPG	1113.5	5.4 (99.5%)	10.7 (99%)	12.8 (98.7%)	11.5 (99%)	32.3 (97.1%)	155.8 (86%)	197.3 (82.3%)	375 (69.2%)	456.4 (56.5%)	595 (44.7%)	1675 (14.5%)	1770 (0%)
	SD	794.3	16.2	24.6	39.8	25.4	44.1	188.7	254.5	395.0	547.6	753.4	1963.4	890.9

## 4 Discussion

The present study provides information on the efficacy of the most commonly used anthelmintics against IS in a horse population in Sardinia. All drugs evaluated in the trial were effective on D14 and no evidence of shortened strongyle ERP was found. As expected, Cyathostominae were predominant (96.4%) in the examined horses, as already reported elsewhere (Peregrine et al., 2014, Molento et al., 2012, Geurden et al., 2014, Larsen et al., 2011). Nonetheless, this study showed no evidence of AR in the examined animals. In Italy, resistance to BZ of small strongyles has been reported for the first time in Northern breedings (Genchi et al., 1992), while AR to BZ and to PYR has been mostly found in central and northern regions (Traversa et al., 2007), and later, other than a wide distribution of resistance to BZ and PYR, a reduced efficacy of IVM was also described in regions of continental Italy (Traversa et al., 2009). A recent survey carried out in ten different Italian locations has shown efficacy of IVM below the 90% ERP threshold on 3 farms on Day 56, and efficacy of MOX below 90% only on 1 study site on Day 84 and from Day 42 onwards in another farm (Geurden et al., 2014).

The present results, although the number of investigated horses was not high, differ from those achieved in continental Italy and other European countries, where drug resistant cyathostomins are spread and the ERP is reduced for FBZ, PYR and, though with a lesser to extent, for IVM and MOX as well. The absence of drug resistance in the examined population of Sardinian horses could be related, besides its insularity (i.e. natural boundaries prevent from the introduction of resistant parasite populations), to the frequency of anthelmintic treatments in the Island. In fact, more than half of the population of Sardinian horses receives a parasiticide less than 2 times per year (Montinaro et al., 2002), thus pressure for selection of resistance is minimal if compared to other management practice. Also, climatic variables could play an important role. In fact, the mild temperate weather of the island allows a high survival rate of environmental L3 all year round, thus preserving the amount of those parasitic stages which do not become in contact with parasiticides and are not selected for resistance, i.e. *refugia (*Nielsen et al., 2014). Although in Italy anthelmintics are sold as veterinary prescription-only medications, a parasitological diagnosis prior to treatment is not required as, for instance, in Denmark where this approach is considered a potential strategy to limit AR in horses (Sallé and Cabaret, 2015).

Similar control plans would be desirable in other settings, in fact a recent questionnaire survey on intestinal worm control practices in horses in Italy showed that only 9.3% of respondents usually dewormed animals after a fecal examination and 61.3% dewormed all horses together (Papini et al., 2015). In fact, as an appropriate use of anthelmintics, along with high-standard practice management, is essential to limit or prevent the development and spread of AR, a prescription-based only treatment would be powerful to reduce the spread of resistant cyathostomins where they are already present and to prevent their introduction in the free regions.

## Conflict of interest statement

The authors declare no conflicts of interest.
